# Real-World Evidence of Porto-Mesenteric Vein Resections with Pancreatectomy and the Development of Predictive Clinical Nomograms for Postoperative Outcomes—An Analysis of 389 Cases: The “Porto-Mesenteric Vein Resection-Indian MulticentrE” (PRIME) Study

**DOI:** 10.1245/s10434-025-17702-1

**Published:** 2025-07-05

**Authors:** Deeksha Kapoor, Manish S. Bhandare, Agam Sharma, Raja Kalayarasan, Monish Karunakaran, Sree Kumar Balasubramanian, Aishwarya Pal, Nagaraj Palankar, D. S. Darshanik, Subhash Soni, Sreenivas Reddy Biravely, ArunKumar Namachivayam, Rajneesh Kumar Singh, Vaibhav Varshney, Adarsh Chaudhary, Sadiq Sikora, Rajesh Gupta, Sanjay Govil, Pradeep Rebala, Biju Pottakkat, Hariharan Ramesh, Vikram A. Chaudhari, Shailesh V. Shrikhande

**Affiliations:** 1 Gastrointestinal and Hepato-Pancreato-Biliary Services, Department of Surgical Oncology, Homi Bhabha National Institute, Tata Memorial Hospital, Parel, Mumbai, Maharashtra India; 2 Department of Surgical Gastroenterology, Lakeshore Hospital and Research Centre, Ernakulam, Kerala India; 3 Department of Surgical Gastroenterology, Jawaharlal Institute of Postgraduate Medical Education and Research, Puducherry, Tamil Nadu India; 4 Department of Surgical Gastroenterology, Asian Institute of Gastroenterology, Hyderabad, Telangana India; 5 Department of Gastrointestinal Surgery and Solid Organ Transplantation, Amrita Institute of Medical Sciences, Edappally, Kochi, Ernakulam, Kerala India; 6 Department of Gastrointestinal Surgery and Liver Transplantation, Manipal Hospitals, Bengaluru, India; 7 Department of Surgical Gastroenterology, All India Institute of Medical Sciences, Jodhpur, Rajasthan India; 8 Department of Surgical Gastroenterology, Sanjay Gandhi Post Graduate Institute of Medical Sciences, Lucknow, Uttar Pradesh India; 9 Bapuji Dental College and Hospital, Davangere, Karnataka India; 10 Division of GI Surgery, GI Oncology, Minimal Access, and Bariatric Surgery, Institute of Digestive and Hepatobiliary Sciences, Gurugram, Haryana India; 11 Department of Gastrointestinal Surgery and Liver Transplantation, Sakra World Hospital, Bengaluru, Karnataka India; 12 Department of Surgical Gastroenterology, Postgraduate Institute of Medical Education and Research, Chandigarh, India; 13 Department of Gastro, Hepatobiliary, Pancreatic Surgery and Liver Transplantation, Apollo Hospital, Bengaluru, Karnataka India

**Keywords:** Porto-mesenteric vein resection, Pancreatectomy, Pancreatic ductal adenocarcinoma, Disease-free survival, Overall survival

## Abstract

**Background:**

With better surgery and chemotherapeutic agents, borderline resectable or locally advanced pancreatobiliary tumours are being treated with curative intent. This study presents real-world evidence of porto-mesenteric vein resections (PVR) with pancreatectomy and generates predictive nomograms for postoperative mortality (POM) and major complications (MC).

**Methods:**

A retrospective multicentre study, including 11 high-volume centres, evaluated patients undergoing PVR. Factors affecting 90-day POM and MC (Clavien-Dindo grades ≥ 3a) were assessed, and predictive nomograms were generated. Overall survival (OS) and disease-free survival (DFS) were estimated for patients with pancreatic ductal adenocarcinoma (PDAC). Cox regression analysis was performed to ascertain factors affecting OS and DFS.

**Results:**

Among 389 patients, POM was 6.4%, and MCs were 32.6%. Charlson comorbidity index > 4, preoperative biliary drainage, preoperative radiotherapy (PRT), segmental PVR, and additional organ resection (AOR) were predictive of POM. The independent predictors of MCs were American Society of Anesthesiologists status 3/4, PRT, and AOR. The generated model had an area under the curve (AUC) of 0.757, cutoff > 1.79 to predict POM, and AUC of 0.669, cutoff > 0.678 for MCs. In the 263 patients with PDAC, the median OS was 25.01 months (95% confidence interval [CI] 21.9–28.11), and DFS was 16.72 months (95% CI 14.56–18.89). Perineural invasion, segmental PVR, and margin positivity predicted worse survival, while completing multi-modality treatment was protective.

**Conclusions:**

The POM and MCs of PVR with pancreatectomy were at par with the world standards. The generated predictive nomograms for POM and MC revealed a good predictive potential. In patients with PDAC, completion of multimodality treatment offers better long-term survival.

**Supplementary Information:**

The online version contains supplementary material available at 10.1245/s10434-025-17702-1.

Tumour abutment or encasement of the porto-mesenteric vein, hepatic artery, or superior mesenteric artery is relatively common in non-metastatic pancreatobiliary tumours, rendering them borderline resectable or locally advanced.^1^ Venous involvement often warrants resection, whereas divestment or resection might be considered for arterial involvement.^2,3^ Although such resections have previously evoked nihilism, advancements in chemotherapy and surgical techniques have made aggressive resections feasible and safer.^1,4–6^ Porto-mesenteric vein resection (PVR) during pancreatectomy for tumours involving the vein is now considered a standard of care following adequate neoadjuvant therapy (NAT), provided R0 resection is deemed achievable, despite the greater risk of complications associated with this procedure.^3,7^ The extent of PVR is guided by both the circumferential and longitudinal extent of the vein involvement. With limited vein involvement, venous sleeve resection with or without patch closure is sufficient. More extensive vein involvement may require segmental resection with end-to-end anastomosis or an interposition graft.^7^

The emerging literature highlights that the number and complexity of pancreatic resections have significantly increased in India over the past two decades, with an increase in vascular resections being a natural consequence.^7–11^ Evaluating the outcomes of these procedures and analysing practice patterns in preoperative therapy and planning are essential. The key goals to achieve after surgery of such complexity are twofold: optimising postoperative outcomes and ensuring timely initiation and completion of systemic treatment to maximise survival benefits.

This study presents real-world data on PVRs with pancreatectomy, focusing on postoperative and survival outcomes. The primary objective was to ascertain the factors predicting 90-day postoperative mortality (POM) and major complications (MC). Secondarily, overall and disease-free survival rates were studied in patients with pancreatic ductal adenocarcinoma (PDAC), and factors impacting survival outcomes were analysed.

## Methods

### Study Design and Patient Selection

In October 2023, 11 high-volume pancreatobiliary centres collaborated for the “Porto-mesenteric vein Resection-Indian Multicenter” (PRIME) study to report real-world data on PVR with pancreatectomy.^12^ Retrospective data, including those of patients who underwent PVR with pancreatectomy, were collected from January 2015 to September 2023. Patients lacking at least three months of follow-up were excluded (Supplementary Fig. 1). The initial institutional review board and ethics committee approvals were obtained from the Tata Memorial Hospital, Mumbai (OIEC/4381/2024/0001), the primary coordinating centre. This study adhered to the Declaration of Helsinki and followed the STROBE guidelines for reporting observational studies.^13,14^

### Data Collection

Data were collected by using a structured Excel spreadsheet, including de-identified information on clinical parameters, preoperative imaging, and treatment details. Surgical data, postoperative outcomes, adjuvant therapy, and follow-up information were collected. Patients who had not visited the clinic in the past 3 months were telephonically contacted for an updated follow-up. After thorough data cleaning, to ensure data accuracy, completeness, and consistency, the primary team contacted the contributing centres for data checks. Duplicates, missing values, outliers, and inconsistencies were communicated to the respective centres and rectified.

### Definitions Deployed in the Study

The resectability of primary pancreatic tumours was defined according to the 2023 National Comprehensive Cancer Network (NCCN) criteria.^2,15^ Porto-mesenteric vein resection types were classified based on the International Study Group of Pancreatic Surgery (ISGPS) criteria: type 1, partial vein excision with primary closure; type 2, partial vein excision with patch closure; type 3, segmental PVR with end-to-end anastomosis; and type 4, segmental PVR with an interposition graft.^2^ Additional organ resection (AOR) was defined as the resection of an organ not typically included in a standard pancreatoduodenectomy (PD) or distal pancreatosplenectomy (DPS).^16^ Postoperative pancreatic fistula, grades B and C (POPF), post-pancreatectomy hemorrhage (PPH), and delayed gastric emptying (DGE) were defined according to ISGPS guidelines.^17–19^ Postoperative complications were graded by using the Clavien-Dindo (CD) classification, and grade ≥ 3a was considered major.^20^ Postoperative mortality was defined as all-cause mortality within 90 days after surgery.^21^ Failure to rescue was defined as failure to salvage a patient after the patient developed MC.^22^ Treatment completion was marked when the patient completed the multimodality regimen planned by the treating institute. The eighth edition of the American Joint Committee on Cancer Staging (AJCC) was used for disease staging.^23,24^ R1 resection was noted if tumor cells were present within 1 mm of the resection margin.^25^ The Charlson’s comorbidity index (CCI) was calculated by using the MDCalc CCI online tool.

Disease recurrence was identified through follow-up imaging (radiological recurrence) and confirmed by biopsy when feasible. Given that many patients received NAT, overall survival (OS) was defined as the time from diagnosis to death or the last follow-up. Disease-free survival (DFS) was measured from the date of curative surgery to recurrence, either death or last follow-up.^26^ If a patient died during follow-up without recurrence, this was considered an endpoint for recurrence-free survival. Patients with missing recurrence or survival data were censored on the date of their last follow-up. The primary outcomes were MC and POM, and predictive nomograms were constructed for these outcome measures. Overall survival and DFS were evaluated in patients undergoing surgery for pancreatic ductal adenocarcinoma (PDAC).

### Statistical Analysis

The baseline characteristics were summarised by using descriptive statistics. Categorical variables are presented as proportions and percentages, with comparisons made using the chi-square test. Normally distributed continuous variables are reported as means with standard deviations, whereas nonparametric continuous variables are expressed as medians with interquartile ranges. Means between two groups were compared by using independent Student’s *t*-tests, and medians were compared using the Mann-Whitney *U* test. The factors influencing the development of MC and POM were analysed by using both univariate and multivariate analyses.

Predictive models for POM and MC were constructed using the β coefficients generated from the multivariate analysis. Nomograms were developed with Python libraries, including statsmodels and simple nomograms. This process involved converting the target variable into integers and adding an intercept to the predictors. After fitting the model, the coefficients were extracted and organised into a DataFrame, which included coefficients for each predictor, the intercept, and a placeholder for the threshold. A Simple Nomogram package was employed to generate and visualise the nomogram. The model’s performance was assessed by plotting a receiver operating characteristic (ROC) curve and calculating the area under the curve (AUC).

Kaplan-Meier curves were plotted for OS and DFS, and log-rank tests were employed to compare the groups. A multivariable Cox proportional hazards analysis assessed factors influencing OS and DFS, adjusting for potential confounders. The results were presented as hazard ratios (HR) with corresponding 95% confidence intervals. Statistical analysis was performed by using IBM SPSS (International Business Machine—Statistical Package for the Social Sciences) v29.0 for Mac and Python v3.11. All tests were two-sided, and statistical significance was set at 5%.

## Results

### Overall Cohort

During the study period, 6150 pancreatectomies were performed, with 422 patients undergoing concomitant PVR (6.86%). Nineteen cases were excluded owing to a lack of 3-month follow-up. Fourteen patients who underwent arterial resection and anastomosis were also excluded (Supplementary Fig. 1). The final cohort included 389 patients, with a mean age of 56.12 ± 12.8 years, comprising 59.1% men and 40.9% women. The most common indication for PVR was PDAC, accounting for 263 (67.6%) cases, followed by pancreatic neuroendocrine tumours (pNET), with 31 (8%) cases (Supplementary Table 1). Of the 389 tumours, 151 (38.8%), 207 (53.2%), and 31 (8%) were classified as RPC, BRPC, and LAPC, respectively. Neoadjuvant therapy was administered to 147 (37.8%) patients, and four patients with pNET received peptide receptor radionuclide therapy with neoadjuvant intent. The centre-wise distribution of cases reporting the main outcome parameters is presented in Supplementary Table 2.

### Surgical Details

The most commonly performed surgeries were PD, 340 (87.4%), followed by DPS, 35 (9%), and total pancreatectomy (TP), 14 (3.6%). Additional organs were resected in 58 (14.9%) patients, with the colon being the most frequently excised organ (38/58). Type 3 ISGPS was the most prevalent PVR, at 189 (48.7%), followed by type 1, 147 (37.9%) (Supplementary Table 1). Six (1.5%) patients underwent venous resection without reconstruction (VROR). In type 2 PVR, the peritoneal and saphenous vein patches were the most commonly utilised. In type 4 PVR, autologous grafts (most often internal jugular veins) were used in 15 of 28 patients, whereas synthetic grafts were employed in 13 of 28 patients. Portal venous clamping times were available for 223 patients, with a median of 20 (range 7–65) min. Ten (2.6%) procedures were performed using the minimal access approach: six robotic PD, one robotic DPS, one laparoscopic PD, and two laparoscopic DPS.

### Postoperative Outcomes

**Fig. 1:**
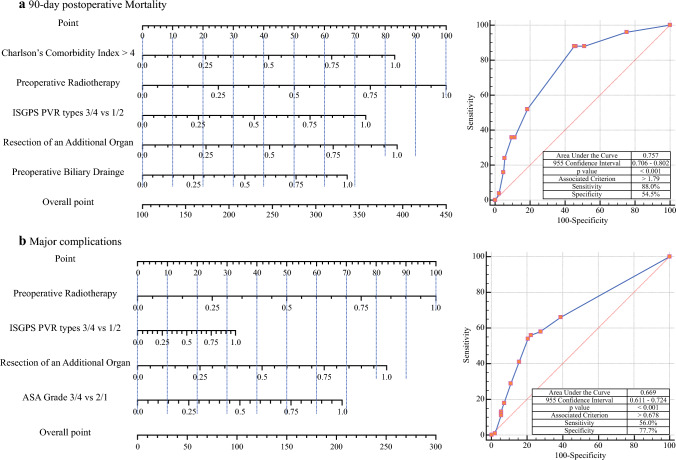
Predictive nomograms and respective receiver operating characteristic curves. **a** 90-day postoperative mortality. **b** Major complications

Major complications occurred in 127 (32.6%) patients, and 25 (6.4%) required re-exploration. The mortality rate at 30 days was 2.8% (11/389), whereas the 90-day POM rate was 6.4% (25/389). Postoperative pancreatic fistula developed in 52 (13.4%) patients, PPH in 47 (12.1%), and DGE in 141 (36.2%). Chyle leaks were observed in 58 (14.9%) patients. Major complications were seen in 28.5% (47/165) of wedge PVR and 35.7% (80/224) of segmental PVR (*p* = 0.116), with the POM rate being higher in segmental PVR at 9.4% (21/224) compared with wedge resections at 2.4% (4/166), *p* = 0.005 (Fig. 1).

### 90-day Postoperative Mortality

Factors significant at the 10% level in the univariate analysis were included in the multivariate analysis. Higher CCI, preoperative biliary drainage (PBD), preoperative radiotherapy (PRT), segmental PVR (ISGPS types 3, 4, and VROR), AOR, and increased operative blood loss were identified as significant risk factors. ROC analysis established a cutoff > 4 for CCI (AUC = 0.711, sensitivity = 68%, specificity = 66.5%) and for operative blood loss > 675 mL (AUC = 0.712, sensitivity = 80%, specificity = 54.5%). In the multivariate analysis, CCI > 4, PBD, PRT, segmental PVR, and AOR independently predicted POM (Table 1).

**Table 1:** Multivariate analysis of factors predicting major complications (Clavien Dindo Grade 3 and above) and 90-day postoperative mortality

Clinical parameter	90-Day postoperative mortality			Major complications		
	Odds ratio	95% Confidence interval	*p*	Odds ratio	95% Confidence interval	*p* value
Charlson’s comorbidity index > 4	5.209	2.033–13.348	*<0.001*			
ASA grade 3 + 4 vs. 2 + 1				1.899	1.101–3.276	*0.021*
Radiologically resectable						
Radiologically borderline resectable				1.000	0.606–1.65	0.999
Radiologically locally advanced				1.538	0.618–3.825	0.355
Preoperative biliary drainage	3.871	1.419–10.563	*0.008*			
Preoperative radiotherapy	6.345	1.887–21.33	*0.003*	2.499	1.122–5.563	*0.025*
ISGPS vein resection 3 + 4 vs. 1 + 2^a^	4.1	1.297–12.963	*0.016*	1.363	0.856–2.172	0.192
Resection of additional organs	5.297	1.726–16.262	*0.004*	3.727	1.971–1.042	*0.037*
Operative blood loss > 675 ml	1.563	0.609–4.014	0.353			
Right-sided vs. left-sided/total pancreatectomy				0.763	0.376–1.55	0.455

*ASA* American society of anesthesiologists; *ISGPS* international study group of pancreatic surgery

^a^Vein resections without reconstruction included with type 3+4 vein resections

*p* values significant at < 0.05 have been italicised

### Model Design

As per the generated ß coefficients, the following model equation was created to predict the POM.

Equation = −6.047 +1.677 (CCI > 4) +1.362 (PBD) + 2.018 (PRT) + 1.482 (PVR 3/4) + 1.691 (AOR).

The ROC analysis generated a cutoff value of >1.79 (AUC = 0.757, sensitivity = 88%, specificity = 54.5%). (Fig. 1a).

### Major Complications

Radiological resectability, ASA grade, PRT, segmental PVR, AOR, and right-sided resection were associated with MCs on univariate analysis, significant at 10%, and were included in the multivariate analysis. American Society of Anaesthesiology (ASA) grade 3/4, PRT, and AOR were identified as independent MC predictors (Table 1). Segmental PVRs were not associated with the development of MCs but with higher rates of POM.

**Table 2:** Multivariable Cox proportional hazard analysis of factors predicting overall survival and disease-free survival in patients undergoing portal vein resection with pancreatectomy for carcinoma pancreas

Clinical parameter	Overall survival			Disease-free survival		
	Hazard ratio	95% Confidence interval	*p*	Hazard ratio	95% Confidence interval	*p*
Positive resection margin	2.424	1.613–3.642	*<0.001*	2.079	1.432–3.02	*<0.001*
Perineural invasion	1.811	1.179–2.784	*0.007*	1.762	1.198–2.594	*0.004*
Right-sided vs. left-sided pancreatic resection	1.693	0.882–3.251	0.114			
Venous resection 3 + 4 vs. 1 + 2^a^	1.531	1.015–2.307	*0.042*	1.600	1.111–2.305	*0.003*
Maximum tumour size (mm)	1.249	1.032–1.51	*0.022*	1.096	0.919–1.307	0.306
Nodal stage 2 vs. 0 + 1	1.345	0.899–2.013	0.149	1.417	0.969–2.074	0.072
Additional organ resection	0.548	0.287–1.045	0.068	0.611	0.375–0.994	*0.047*
Completion of multimodality treatment	0.437	0.293–0.651	*<0.001*	0.567	0.391–0.82	*0.003*

^a^Vein resections without reconstruction included with type 3+4 vein resections

*p* values significant at < 0.05 have been italicised

### Model Design

Based on the generated ß coefficients, the ensuing model equation was formulated to predict the MCs:

Equation = −1.293 + 0.683 (ASA Grade 3/4) + 0.995 (PRT) + 0.327 (PVR 3/4) + 0.831 (AOR).

The ROC analysis generated a cutoff value of >0.678 (AUC = 0.669, sensitivity = 56%, specificity = 77.7%) (Fig. 1b).

### Failure to Rescue

The failure-to-rescue rate (FTR) was 19.7% (25/127). A higher FTR rate was associated with CCI > 4 (13.4% vs. 6.3%, *p* = 0.006), PBD (68% vs. 45.1%, *p* = 0.047), and segmental PVR (84% vs. 57.8%, *p* = 0.02) (Supplementary Table 3).

### Outcomes in Pancreatic Cancer

Of 389 patients, 263 underwent surgery for PDAC, and 125 received NAT. The most common regimens were mFOLFIRINOX (*n* = 76) and gemcitabine plus nab-paclitaxel (*n* = 28), followed by gemcitabine-based chemotherapy. The number of NAT cycles ranged from 2 to 12, with a median of 4 cycles. The mean tumour size was 3.11 cm, and R1 resection was performed in 69 (26.2%) patients. A complete pathological response was achieved in three patients. The stage distribution was as follows: stage 1, 73 (27.8%); stage 2, 121 (46%); and stage 3, 69 (26.2%). Planned multimodality treatment was completed in 153 patients (58.2%). The most common reasons for not completing adjuvant therapy were poor general status (*n* = 15), chemotherapy-related toxicity (*n* = 12), patient dropout (*n* = 12), and postoperative MCs (*n* = 8).

#### Overall Survival

**Fig. 2:**
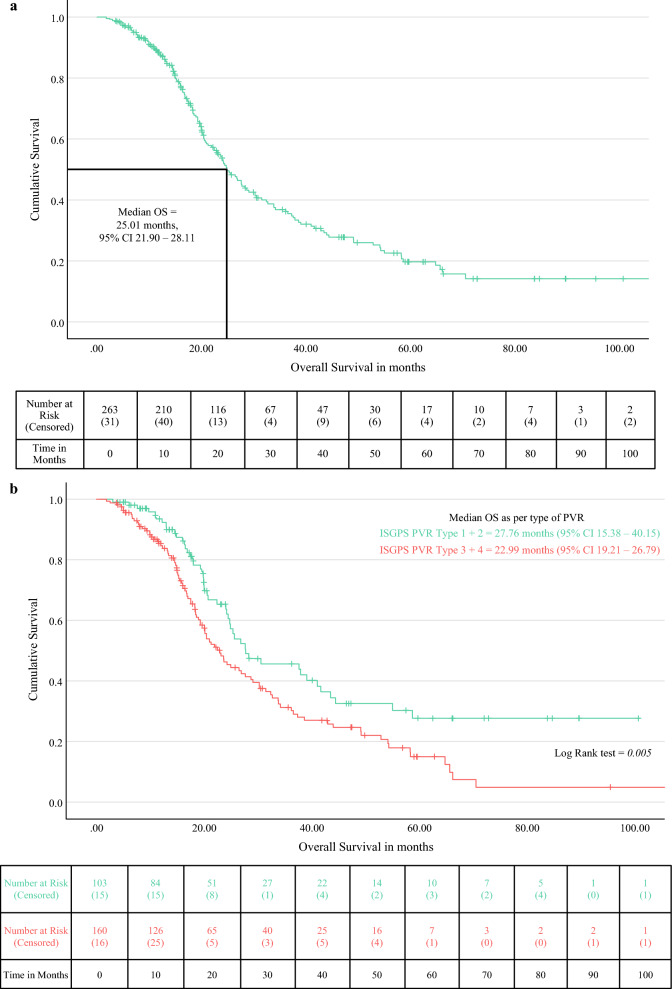

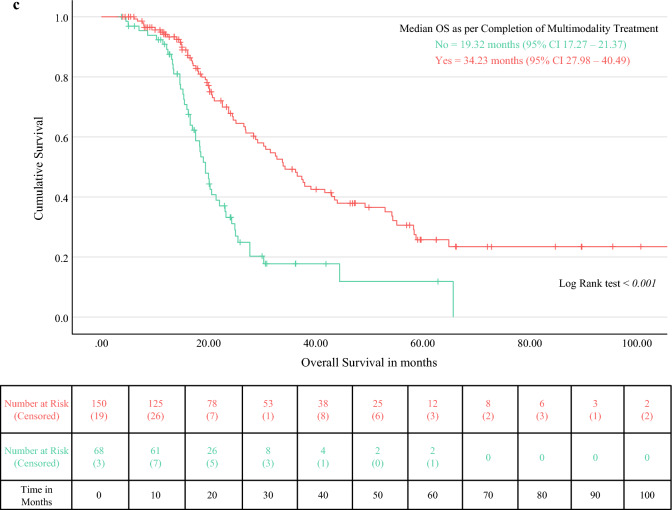
**a** Kaplan-Meier curve for overall survival of patients undergoing portal vein resection with pancreatectomy for carcinoma pancreas. **b** Log-rank test for overall survival of patients undergoing ISGPS PVR type 1/2 vs 3/4. **c** Log-rank test for overall survival of patients completing vs not completing the planned multimodality treatment

The median OS for PDAC was 25.01 months (95% CI 21.9–28.11) (Fig. 2a). The median follow-up duration, estimated using reverse Kaplan-Meier analysis, was 36.24 months (95% CI 23.73–48.75). The 1-year, 3-year, and 5-year survival rates were 72.2%, 20.9%, and 6.5%, respectively (Fig. 2a). Cox proportional hazards analysis identified margin positivity, perineural invasion, segmental PVR, and larger tumour size as independent predictors of poor OS (Table 2; Fig. 2b). Conversely, when indicated, AOR and completion of the planned multimodal treatment were protective (Fig. 2c). In subgroup analysis, OS was not significantly different between BRPC and LAPC (log-rank test, *p* = 0.404). Eighty-three patients radiologically classified as RPC ultimately underwent PVR: nine had received NAT, six because of high Ca19-9 levels, six because of anticipated PVR, and two because of suspected R1 resection. The survival advantage of NAT could not be demonstrated (HR 0.993, *p* = 0.967).

#### Disease-Free Survival

**Fig. 3:**
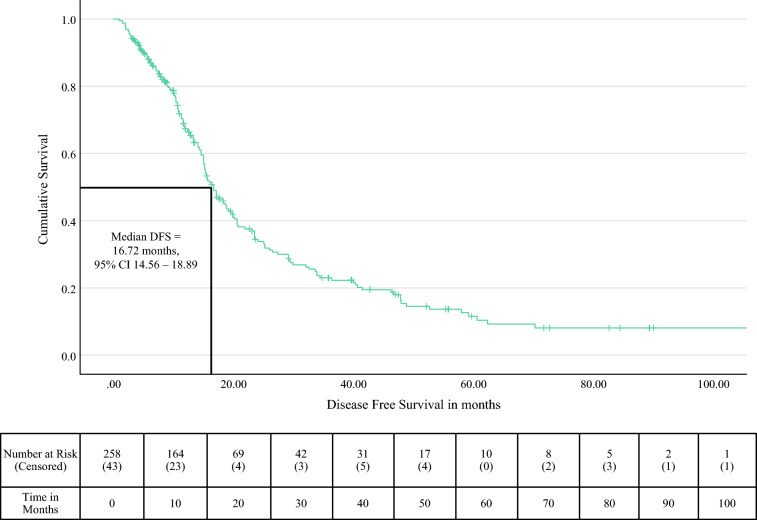
Kaplan-Meier curve for disease-free survival of patients undergoing portal vein resection with pancreatectomy for carcinoma pancreas

The median DFS was 16.72 months (95% CI 14.56–18.89), with the 1-year, 3-year, and 5-year DFS rates at 51.9%, 13.2%, and 3.8% respectively (Fig. 3). The earliest recurrence occurred at 1.4 months. Distant recurrences alone were noted in 24.2% of patients, isolated local recurrences in 15.7%, and combined distant and local recurrences in 10.1%. Margin-positive resection, segmental PVR, and perineural invasion were independent predictors of poorer DFS (Table 2). Similar to OS, the completion of multimodal treatment and AOR proved to be protective (Fig. 3).

## Discussion

The PRIME study reported a 90-day POM of 6.4% and an MC of 32.6%. The 30-day mortality rate was 2.8%. CCI > 4, PBD, PRT, AOR, and segmental PVR were identified as independent predictors of POM. ASA grade 3/4, AOR, and PRT were also found to be independent predictors of MCs. The failure-to-rescue rate stood at 19.7%, which was higher in patients with CCI > 4 and those with segmental PVR. These variables were incorporated into predictive nomograms to illustrate their complex interplay and collective impact on POM and MCs.

Historically, the incidence of pancreatic tumours in India has been considered low.^27–29^ However, pancreatic resections have become more common, given the growing population, increasing disease incidence, and broader acceptance of multimodality treatments. This is likely to drive an increase in complex procedures, such as vascular and multivisceral resections, especially at specialised pancreatobiliary centres. The MC and POM rates in this study were consistent with international reports on pancreatectomy with PVR.^4,30,31^ A recent study established benchmarks for outcomes of PD with PVR: in-hospital mortality at 2.8%, POPF rate at 13.4%, and 5-year survival in PDAC patients at 6.5%.^30^ The findings of this study align with these benchmarks. While several studies have compared morbidity rates between pancreatectomy with and without PVR, only a few studies have reported a comprehensive assessment of factors predicting postoperative MC and POM, and none have developed or reported a predictive nomogram, which is a novel contribution of the present study.

Increased complications in patients undergoing vascular resection have been documented previously.^4^ However, when indicated, PVR has become the standard of care, offering a significant survival advantage over palliative chemoradiotherapy.^32–34^ Several crucial differences emerge when comparing the results of this study with those of a previous large series. Although segmental PVRs are generally regarded as high-risk surgeries linked to elevated complication rates, the current study did not demonstrate a significantly higher MC rate (CD Grade 3 and 4) (35.7% vs. 28.5%, *p* = 0.116) in patients undergoing segmental PVR. Segmental PVR was associated with increased POM (9.4% vs. 2.4%, *p* = 0.005), indicating worse failure-to-rescue rates (84% vs. 16%, *p* = 0.02) (Supplementary Table 3). This contrasts with the findings of Greon et al., who documented increased complications with segmental PVR.^35,36^ Other studies, such as that by Ravikumar et al., provide conflicting evidence, showing no statistically significant impact of segmental PVRs on POM and survival.^7,37^ Notably, types 2 and 4 PVRs were performed less frequently in the current series, accounting for 4.6% and 7.2% of cases, respectively. The choice to perform a type 3 PVR or to opt for a less extensive resection, such as patch closure or horizontal venorrhaphy, is largely surgeon-dependent and requires a nuanced understanding of the venous anatomy. Complications associated with segmental PVR may not be solely attributable to the nature of vein resection. Additional factors, such as splenic vein ligation, postoperative congestive gastropathy, and venous thrombosis, which are more prevalent with such resections, are likely to contribute to the heightened risk.

Extended pancreatectomy often involves resecting an additional organ; the colon was the most frequently resected organ in this study.^16^ During lengthy vein excisions, the right colon is particularly vulnerable to devascularisation, which sometimes necessitates its removal. While extended pancreatectomy is generally associated with higher mortality and morbidity rates, other studies have reported comparable outcomes.^10,16,38,39^ Preoperative biliary drainage was associated with increased POM and FTR rates. Although the cohort included distal pancreatosplenectomies, 354 (91%) patients underwent PD or total pancreatectomies. Ten minimal access procedures were performed. However, evidence suggests that, in high-volume centres, results are not worse with the robotic or laparoscopic approach.^40,41^

This study also highlighted increased MCs and POM in patients receiving radiation as part of the neoadjuvant protocol. Traditionally, NAT has been linked to reduced morbidity and a significant decrease in POPF.^42^ However, the current findings indicated that while NAT itself was not a significant predictor, PRT was significantly associated with higher MCs and POM. Although this study did not differentiate among the various PRT modalities, doses, and regimens, the absolute benefit of PRT remains debatable.^43^

Successful vascular resection during pancreatectomy relies on a thorough understanding of both venous and arterial anatomy, not only of the pancreas but also of the adjacent organs. A comprehensive grasp of the disease process and careful patient selection are crucial to ensure that the benefits of such high-risk surgery outweigh the inherent morbidity. A key strength of this study is the development of predictive clinical nomograms for POM and MC, which may serve as reliable tools for forecasting outcomes. The models exhibited robust predictive accuracy, as evidenced by the high AUC and concordance (Fig. 1a and b). This model synthesises diverse information into a single predictive framework, offering personalised risk assessment for individual patients. This tailored approach can enhance both short- and long-term outcomes by identifying high-risk patients who might benefit from targeted interventions, such as extensive prehabilitation or treatment sequencing, to allow total neoadjuvant therapy. While this study is the first to comprehensively report on factors predicting postoperative complications using predictive models, the relatively small number of outcome events limits the sample size, preventing its division into training and validation sets, which represents a potential limitation of the study.

In patients with PDAC, the median OS was 25.01 months, and the DFS was 16.72 months, consistent with the outcomes reported in recent studies.^35,44,45^ Segmental PVR was associated with worse OS and DFS, as were R1 resection and perineural invasion. Furthermore, increasing tumour size correlated with diminished OS, aligning with previously published reports.^36^ This study linked a higher nodal stage to reduced DFS, although the association did not achieve statistical significance. While nodal involvement is well established as an adverse prognostic factor, much of the available data predates the widespread adoption of mFOLFIRINOX as the standard treatment for PDAC.^46–48^ The R1 resection rates in this study (approximately 30%) were lower than those reported in the literature, possibly due to differing reporting standards across continents.^30,49^ Such rates, even following vascular resection, indicate an aggressive tumour biology that is difficult to predict preoperatively. Distinguishing actual vein invasion from peritumoral fibrosis or inflammation remains a significant challenge in preoperative imaging and surgical assessments. Studies have reported actual vein involvement in 30–45% of resected specimens, acknowledged as a strong adverse prognostic indicator.^4,37,50–52^

Paradoxically, NAT was not associated with improved OS in this study, mirroring a meta-analysis published by Filho et al.^49^ The NAT regimens employed were highly variable and differed in the number of cycles administered, including radiotherapy. Completing planned multimodal treatment emerged as a strong predictor of improved OS and DFS. No survival difference was observed when focusing solely on radiological BRPC or LAPC (log-rank test, *p* = 0.404), as suggested by previously published literature.^53–55^ The intent of NAT in BRPC and LAPC is to increase margin-negative resections, minimise futile surgery in nonresponders and improve survival. Neoadjuvant therapy tends to select patients most likely to benefit from aggressive resection rather than truly downstaging them, which is also reflected in this study. This underscores the paradigm shift toward prognosis-based resectability, wherein surgical candidacy in BRPC or LAPC is increasingly determined by tumour biology—assessed through serologic markers, such as CA 19-9 response—and patient performance status rather than anatomical resectability criteria alone.^54–57^ Even in the present study, radiological resectability did not correlate with OS or DFS. BRPC and LAPC represent heterogeneous populations with little prognostic difference but significant inter-observer variability.^58–60^ More than two-thirds of the patients undergoing PVR were anatomically resectable tumours, highlighting the limitation of the current anatomic resectability criteria. As an extension, this is why many patients undergoing PVR did not receive NAT.^54,58^ Vascular involvement is increasingly considered a technical or topographical challenge rather than a definitive marker of tumour aggression.^8,61,62^ This highlights the limitations of an anatomy-based prognostic approach in pancreatic cancer, particularly as the disease is increasingly recognised as systemic from the outset.^45,54,60,63^

This study has several limitations, primarily due to its retrospective design, which may introduce information bias in data collection and outcome classification. However, most collaborating centres utilise electronic medical records and maintain prospective databases, enhancing the data's reliability. Patients with incomplete records or follow-ups of fewer than 3 months were excluded. Additionally, variations in treatment and operative protocols across participating institutions related to NAT, surgical planning, and standards may have introduced unaccounted confounders into this observational study. The histopathological reporting protocols can differ among institutions. Nevertheless, standardised definitions and detailed reporting instructions were provided to all participating centres, and pathology-related data points were harmonised according to the 8th edition of the AJCC. However, a comprehensive evaluation of the histopathological involvement of the resected veins, widely considered a critical prognostic factor, was not available.^4,36,45–50^

## Conclusions

This study provides real-world insights into PVR with pancreatectomy in India, reporting MCs and POM consistent with global standards. It also introduces predictive nomograms for MC and POM with a reasonable accuracy. The median OS, at 25.01 months, has significantly improved compared with the 14–19 months reported in earlier studies from the region. This enhancement in survival rates is primarily attributed to the improved efficacy of chemotherapy, widespread implementation of multimodality treatment, and better surgical outcomes following aggressive resections in expert centres.

## Supplementary Information

Below is the link to the electronic supplementary material.

Supplementary file1 (DOCX 236 KB)

## References

[1] Alemi F, Rocha FG, Helton WS, Biehl T, Alseidi A. Classification and techniques of en bloc venous reconstruction for pancreaticoduodenectomy. *HPB*. 2016;18(10):827–34. 10.1016/j.hpb.2016.05.015. 27506994 10.1016/j.hpb.2016.05.015 PMC5061022

[2] Bockhorn M, Uzunoglu FG, Adham M, et al. Borderline resectable pancreatic cancer: a consensus statement by the international study group of pancreatic surgery (ISGPS). *Surgery*. 2014;155(6):977–88. 10.1016/j.surg.2014.02.001. 24856119 10.1016/j.surg.2014.02.001

[3] Toomey P, Hernandez J, Morton C, et al. Resection of portovenous structures to obtain microscopically negative margins during pancreaticoduodenectomy for pancreatic adenocarcinoma is worthwhile. *Am Surg*. 2009;75(9):804–10. 10.1177/000313480907500911. 19774952 10.1177/000313480907500911

[4] Giovinazzo F, Turri G, Katz MH, Heaton N, Ahmed I. Meta-analysis of benefits of portal–superior mesenteric vein resection in pancreatic resection for ductal adenocarcinoma. *Br J Surg*. 2016;103(3):179–91. 10.1002/bjs.9969. 26663252 10.1002/bjs.9969

[5] Macedo FI, Ryon E, Maithel SK, et al. Survival outcomes associated with clinical and pathological response following neoadjuvant FOLFIRINOX or gemcitabine/nab-paclitaxel chemotherapy in resected pancreatic cancer. *Ann Surg*. 2019;270(3):400–13. 10.1097/SLA.0000000000003468. 31283563 10.1097/SLA.0000000000003468 PMC9634701

[6] Hank T, Klaiber U, Hinz U, et al. Oncological outcome of conversion surgery after preoperative chemotherapy for metastatic pancreatic cancer. *Ann Surg*. 2023;277(5):e1089–98. 10.1097/SLA.0000000000005481. 35758505 10.1097/SLA.0000000000005481 PMC10082047

[7] Ravikumar R, Sabin C, Hilal MA, et al. Portal vein resection in borderline resectable pancreatic cancer: a United Kingdom multicenter study. *J Am Coll Surg*. 2014;218(3):401–11. 10.1016/j.jamcollsurg.2013.11.017. 24484730 10.1016/j.jamcollsurg.2013.11.017

[8] Shrikhande SV, Shinde RS, Chaudhari VA, et al. Twelve hundred consecutive pancreato-duodenectomies from single centre: impact of centre of excellence on pancreatic cancer surgery across India. *World J Surg*. 2020;44(8):2784–93. 10.1007/s00268-019-05235-0. 31641837 10.1007/s00268-019-05235-0

[9] Kapoor D, Perwaiz A, Singh A, Kumar AN, Chaudhary A. Factors predicting 30-day mortality after pancreaticoduodenectomy—the impact of elevated aspartate aminotransferase. *Langenbecks Arch Surg*. 2023;408(1):130. 10.1007/s00423-023-02865-w. 36991246 10.1007/s00423-023-02865-w

[10] Chaudhari VA, Kunte AR, Chopde AN, et al. Evolution and improved outcomes in the era of multimodality treatment for extended pancreatectomy. *BJS Open*. 2024;8(4):zrae065. 10.1093/bjsopen/zrae065. 39088732 10.1093/bjsopen/zrae065 PMC11293468

[11] Kapoor D, Desiraju Y, Chaudhari VA, et al. Validation and optimisation of the ISGPS risk classification for postoperative pancreatic fistula after pancreatoduodenectomy for periampullary tumours. *Ann Surg*. 2024. 10.1097/SLA.0000000000006485. 39140617 10.1097/SLA.0000000000006485

[12] Schmidt CM. Effect of hospital volume, surgeon experience, and surgeon volume on patient outcomes after pancreaticoduodenectomy: a single-institution experience. *Arch Surg*. 2010;145(7):634. 10.1001/archsurg.2010.118. 20644125 10.1001/archsurg.2010.118

[13] Goodyear MDE, Krleza-Jeric K, Lemmens T. The declaration of Helsinki. *BMJ*. 2007;335(7621):624–5. 10.1136/bmj.39339.610000.BE. 17901471 10.1136/bmj.39339.610000.BE PMC1995496

[14] Von Elm E, Altman DG, Egger M, Pocock SJ, Gøtzsche PC, Vandenbroucke JP. The strengthening the reporting of observational studies in epidemiology (STROBE) statement: guidelines for reporting observational studies. *Int J Surg*. 2014;12(12):1495–9. 10.1016/j.ijsu.2014.07.013. 25046131 10.1016/j.ijsu.2014.07.013

[15] Conroy T, Pfeiffer P, Vilgrain V, et al. Pancreatic cancer: ESMO clinical practice guideline for diagnosis, treatment and follow-up. *Ann Oncol*. 2023;34(11):987–1002. 10.1016/j.annonc.2023.08.009. 37678671 10.1016/j.annonc.2023.08.009

[16] Hartwig W, Vollmer CM, Fingerhut A, et al. Extended pancreatectomy in pancreatic ductal adenocarcinoma: definition and consensus of the international study group for pancreatic surgery (ISGPS). *Surgery*. 2014;156(1):1–14. 10.1016/j.surg.2014.02.009. 24856668 10.1016/j.surg.2014.02.009

[17] Bassi C, Marchegiani G, Dervenis C, et al. The 2016 update of the international study group (ISGPS) definition and grading of postoperative pancreatic fistula: 11 years after. *Surgery*. 2017;161(3):584–91. 10.1016/j.surg.2016.11.014. 28040257 10.1016/j.surg.2016.11.014

[18] Wente MN, Bassi C, Dervenis C, et al. Delayed gastric emptying (DGE) after pancreatic surgery: a suggested definition by the international study group of pancreatic surgery (ISGPS). *Surgery*. 2007;142(5):761–8. 10.1016/j.surg.2007.05.005. 17981197 10.1016/j.surg.2007.05.005

[19] Wente MN, Veit JA, Bassi C, et al. Postpancreatectomy hemorrhage (PPH)–an international study group of pancreatic surgery (ISGPS) definition. *Surgery*. 2007;142(1):20–5. 10.1016/j.surg.2007.02.001. 17629996 10.1016/j.surg.2007.02.001

[20] Clavien PA, Barkun J, De Oliveira ML, et al. The Clavien-Dindo classification of surgical complications: five-year experience. *Ann Surg*. 2009;250(2):187–96. 10.1097/SLA.0b013e3181b13ca2. 19638912 10.1097/SLA.0b013e3181b13ca2

[21] Mise Y, Vauthey JN, Zimmitti G, et al. Ninety-day postoperative mortality is a legitimate measure of hepatopancreatobiliary surgical quality. *Ann Surg*. 2015;262(6):1071–8. 10.1097/SLA.0000000000001048. 25590497 10.1097/SLA.0000000000001048 PMC4633391

[22] Gleeson EM, Pitt HA, TaraM M, et al. Failure to rescue after pancreatoduodenectomy: a transatlantic analysis. *Ann Surg*. 2021;274(3):459–66. 10.1097/SLA.0000000000005000. 34132696 10.1097/SLA.0000000000005000

[23] Kamarajah SK, Burns WR, Frankel TL, Cho CS, Nathan H. Validation of the American joint commission on cancer (AJCC) 8th edition staging system for patients with pancreatic adenocarcinoma: a surveillance, epidemiology and end results (SEER) analysis. *Ann Surg Oncol*. 2017; 24(7): 2023–30. 10.1245/s10434-017-5810-x. 10.1245/s10434-017-5810-x 28213792

[24] Amin MB, Greene FL, Edge SB, et al. The eighth edition AJCC cancer staging manual continuing to build a bridge from a population‐based to a more “personalized” approach to cancer staging. *CA A Cancer J Clin*. 2017; 67(2): 93–9. 10.3322/caac.21388 10.3322/caac.21388 28094848

[25] Campbell PF, Cairns DA, Duthie DF, Feakins PR. Dataset for histopathological reporting of carcinomas of the pancreas, ampulla of Vater and common bile duct October 2019.

[26] Gyawali B, Eisenhauer E, Tregear M, Booth CM. Progression-free survival: it is time for a new name. *Lancet Oncol*. 2022;23(3):328–30. 10.1016/S1470-2045(22)00015-8. 35240080 10.1016/S1470-2045(22)00015-8

[27] Gaidhani RH, Balasubramaniam G. An epidemiological review of pancreatic cancer with special reference to India. *IJMS*. 2021;73:99–109. 10.25259/IJMS_92_2020.

[28] Takiar R, Nadayil D, Nandakumar A. Projections of number of cancer cases in India (2010–2020) by cancer groups. *Asian Pac J Cancer Prev*. 2010;11(4):1045–9. 21133622

[29] Dhir V, Mohandas KM. Epidemiology of digestive tract cancers in India IV. Gall bladder and pancreas. *Indian J Gastroenterol*. 1999;18(1):24–8. 10063743

[30] Raptis DA, Sánchez-Velázquez P, Machairas N, et al. Defining benchmark outcomes for pancreatoduodenectomy with portomesenteric venous resection. *Ann Surg*. 2020;272(5):731–7. 10.1097/SLA.0000000000004267. 32889866 10.1097/SLA.0000000000004267

[31] Ren L, Jäger C, Schorn S, et al. Arterial resection for pancreatic cancer: feasibility and current standing in a high-volume center. *Ann Surg Open*. 2023;4(3):e302. 10.1097/AS9.0000000000000302. 37746627 10.1097/AS9.0000000000000302 PMC10513225

[32] Doi R, Imamura M, Hosotani R, et al. Surgery versus radiochemotherapy for resectable locally invasive pancreatic cancer: final results of a randomized multi-institutional trial. *Surg Today*. 2008;38(11):1021–8. 10.1007/s00595-007-3745-8. 18958561 10.1007/s00595-007-3745-8

[33] Lygidakis NJ, Singh G, Bardaxoglou E, et al. Mono-bloc total spleno-pancreaticoduodenectomy for pancreatic head carcinoma with portal-mesenteric venous invasion. A prospective randomized study. *Hepatogastroenterology*. 2004;51(56):427–33. 15086174

[34] Wang C, Wu H, Xiong J, et al. Pancreaticoduodenectomy with vascular resection for local advanced pancreatic head cancer: a single center retrospective study. *J Gastrointest Surg*. 2008;12(12):2183–90. 10.1007/s11605-008-0621-9. 18683009 10.1007/s11605-008-0621-9

[35] Groen JV, Michiels N, Van Roessel S, et al. Venous wedge and segment resection during pancreatoduodenectomy for pancreatic cancer: impact on short- and long-term outcomes in a nationwide cohort analysis. *Br J Surg*. 2021;109(1):96–104. 10.1093/bjs/znab345. 34791069 10.1093/bjs/znab345 PMC10364765

[36] Groen JV, Stommel MWJ, Sarasqueta AF, et al. Surgical management and pathological assessment of pancreatoduodenectomy with venous resection: an international survey among surgeons and pathologists. *HPB*. 2021;23(1):80–9. 10.1016/j.hpb.2020.04.015. 32444267 10.1016/j.hpb.2020.04.015

[37] Ravikumar R, Sabin C, Abu Hilal M, et al. Impact of portal vein infiltration and type of venous reconstruction in surgery for borderline resectable pancreatic cancer. *Br J Surg*. 2017;104(11):1539–48. 10.1002/bjs.10580. 28833055 10.1002/bjs.10580

[38] Stoop TF, Seelen LWF, ’T Land Van FR, et al. Nationwide use and outcome of surgery for locally advanced pancreatic cancer following induction chemotherapy. *Ann Surg Oncol*. 2024;31(4):2640–53. 10.1245/s10434-023-14650-6. 38105377 10.1245/s10434-023-14650-6

[39] Mitra A, Pai E, Dusane R, et al. Extended pancreatectomy as defined by the ISGPS: useful in selected cases of pancreatic cancer but invaluable in other complex pancreatic tumors. *Langenbecks Arch Surg*. 2018. 10.1007/s00423-018-1653-6. 29362882 10.1007/s00423-018-1653-6

[40] Napoli N, Kauffmann EF, Ginesini M, et al. Robotic versus open pancreatoduodenectomy with vein resection and reconstruction: a propensity score-matched analysis. *Ann Surg Open*. 2024;5(2):e409. 10.1097/AS9.0000000000000409. 38911629 10.1097/AS9.0000000000000409 PMC11191888

[41] Napoli N, Cacace C, Kauffmann EF, et al. The PD-ROBOSCORE: a difficulty score for robotic pancreatoduodenectomy. *Surgery*. 2023;173(6):1438–46. 10.1016/j.surg.2023.02.020. 36973127 10.1016/j.surg.2023.02.020

[42] Xue K, Huang X, Zhao P, Zhang Y, Tian B. Perioperative and long-term survival outcomes of pancreatectomy with arterial resection in borderline resectable or locally advanced pancreatic cancer following neoadjuvant therapy: a systematic review and meta-analysis. *Int J Surg*. 2023;109(12):4309–21. 10.1097/JS9.0000000000000742. 38259002 10.1097/JS9.0000000000000742 PMC10720779

[43] Hall WA, Dawson LA, Hong TS, et al. Value of neoadjuvant radiation therapy in the management of pancreatic adenocarcinoma. *J Clin Oncol*. 2021;39(34):3773–7. 10.1200/JCO.21.01220. 34623894 10.1200/JCO.21.01220 PMC8608256

[44] Malleo G, Casciani F, Lionetto G, et al. Resection to exploration ratios and associated outcomes in patients with pancreatic ductal adenocarcinoma. *Ann Surg*. 2024. 10.1097/SLA.0000000000006197. 38214158 10.1097/SLA.0000000000006197

[45] Crippa S, Malleo G, Mazzaferro V, et al. Futility of up-front resection for anatomically resectable pancreatic cancer. *JAMA Surg*. 2024;159(10):1139. 10.1001/jamasurg.2024.2485. 39046713 10.1001/jamasurg.2024.2485 PMC11270270

[46] Shimada K, Sano T, Sakamoto Y, Kosuge T. Clinical implications of combined portal vein resection as a palliative procedure in patients undergoing pancreaticoduodenectomy for pancreatic head carcinoma. *Ann Surg Oncol*. 2006;13(12):1569–78. 10.1245/s10434-006-9143-4. 17009145 10.1245/s10434-006-9143-4

[47] Sperti C, Pasquali C, Piccoli A, Pedrazzoli S. Survival after resection for ductal adenocarcinoma of the pancreas. *Br J Surg*. 2005;83(5):625–31. 10.1002/bjs.1800830512. 10.1002/bjs.1800830512 8689203

[48] Murakami Y, Uemura K, Sudo T, et al. Benefit of portal or superior mesenteric vein resection with adjuvant chemotherapy for patients with pancreatic head carcinoma. *J Surg Oncol*. 2013;107(4):414–21. 10.1002/jso.23229. 22886567 10.1002/jso.23229

[49] Filho JELP, Tustumi F, Coelho FF, et al. The impact of venous resection in pancreatoduodectomy: a systematic review and meta-analysis. *Medicine*. 2021;100(40):e27438. 10.1097/MD.0000000000027438. 34622858 10.1097/MD.0000000000027438 PMC8500612

[50] Fukuda S. Significance of the depth of portal vein wall invasion after curative resection for pancreatic adenocarcinoma. *Arch Surg*. 2007;142(2):172. 10.1001/archsurg.142.2.172. 17309969 10.1001/archsurg.142.2.172

[51] Han SS, Park SJ, Kim SH, et al. Clinical significance of portal-superior mesenteric vein resection in pancreatoduodenectomy for pancreatic head cancer. *Pancreas*. 2012;41(1):102–6. 10.1097/MPA.0b013e318221c595. 21775914 10.1097/MPA.0b013e318221c595

[52] Yekebas EF, Bogoevski D, Cataldegirmen G, et al. En bloc vascular resection for locally advanced pancreatic malignancies infiltrating major blood vessels: perioperative outcome and long-term survival in 136 patients. *Ann Surg*. 2008;247(2):300–9. 10.1097/SLA.0b013e31815aab22. 18216537 10.1097/SLA.0b013e31815aab22

[53] Farnes I, Kleive D, Verbeke CS, et al. Resection rates and intention-to-treat outcomes in borderline and locally advanced pancreatic cancer: real-world data from a population-based, prospective cohort study (NORPACT-2). *BJS Open*. 2023;7(6):zrad137. 38155512 10.1093/bjsopen/zrad137 PMC10755199

[54] Boggi U, Kauffmann EF, Napoli N, et al. REDISCOVER guidelines for borderline-resectable and locally advanced pancreatic cancer: management algorithm, unanswered questions, and future perspectives. *Updates Surg*. 2024;76(5):1573–91. 10.1007/s13304-024-01860-0. 38684573 10.1007/s13304-024-01860-0 PMC11455680

[55] Ventin M, Ferrone CR. REDISCOVER the change in surgical management of pancreatic cancer from anatomy to biology. *Updates Surg*. 2024;76(5):1569–71. 10.1007/s13304-024-01956-7. 39150628 10.1007/s13304-024-01956-7

[56] Oba A, Croce C, Hosokawa P, et al. Prognosis based definition of resectability in pancreatic cancer: a road map to new guidelines. *Ann Surg*. 2022;275(1):175–81. 10.1097/SLA.0000000000003859. 32149822 10.1097/SLA.0000000000003859

[57] Oba A, Del Chiaro M, Fujii T, et al. Conversion surgery for locally advanced pancreatic cancer: a position paper by the study group at the joint meeting of the international association of pancreatology (IAP) & Japan pancreas society (JPS) 2022. *Pancreatology*. 2023;23(6):712–20. 10.1016/j.pan.2023.06.005. 37336669 10.1016/j.pan.2023.06.005

[58] Giannone F, Capretti G, Abu Hilal M, et al. Resectability of pancreatic cancer is in the eye of the observer: a multicenter, blinded, prospective assessment of interobserver agreement on NCCN resectability status criteria. *Ann Surg Open*. 2021;2(3):e087. 10.1097/AS9.0000000000000087. 37635813 10.1097/AS9.0000000000000087 PMC10455302

[59] Reames BN, Blair AB, Krell RW, et al. Management of locally advanced pancreatic cancer: results of an international survey of current practice. *Ann Surg*. 2021;273(6):1173–81. 10.1097/SLA.0000000000003568. 31449138 10.1097/SLA.0000000000003568

[60] Ferrone CR, Marchegiani G, Hong TS, et al. Radiological and surgical implications of neoadjuvant treatment With FOLFIRINOX for locally advanced and borderline resectable pancreatic cancer. *Ann Surg*. 2015;261(1):12–7. 10.1097/SLA.0000000000000867. 25599322 10.1097/SLA.0000000000000867 PMC4349683

[61] Rehders A, Stoecklein NH, Güray A, Riediger R, Alexander A, Knoefel WT. Vascular invasion in pancreatic cancer: tumor biology or tumor topography? *Surgery*. 2012;152(3):S143–51. 10.1016/j.surg.2012.05.012. 22766363 10.1016/j.surg.2012.05.012

[62] Nakao A, Kanzaki A, Fujii T, et al. Correlation between radiographic classification and pathological grade of portal vein wall invasion in pancreatic head cancer. *Ann Surg*. 2012;255(1):103–8. 10.1097/SLA.0b013e318237872e. 22156923 10.1097/SLA.0b013e318237872e

[63] Boggi U, Kauffmann E, Napoli N, et al. REDISCOVER international guidelines on the perioperative care of surgical patients with borderline-resectable and locally advanced pancreatic cancer. *Ann Surg*. 2024. 10.1097/SLA.0000000000006248. 38407228 10.1097/SLA.0000000000006248 PMC11161250

