# Supplementary material for: Real-World Evidence of Porto-Mesenteric Vein Resections with Pancreatectomy and the Development of Predictive Clinical Nomograms for Postoperative Outcomes—An Analysis of 389 Cases: The “Porto-Mesenteric Vein Resection-Indian MulticentrE” (PRIME) Study
Source: Ann Surg Oncol. 2025 Jul 5;32(10):7410–21. doi: 10.1245/s10434-025-17702-1 (PMC12454574; doi:10.1245/s10434-025-17702-1)
Supplement: Supplementary file 1 — Supplementary file1 (DOCX 236 KB) [file 10434_2025_17702_MOESM1_ESM.docx]

**Supplementary Tables**

**Supplementary Figure 1** – Study Flowchart


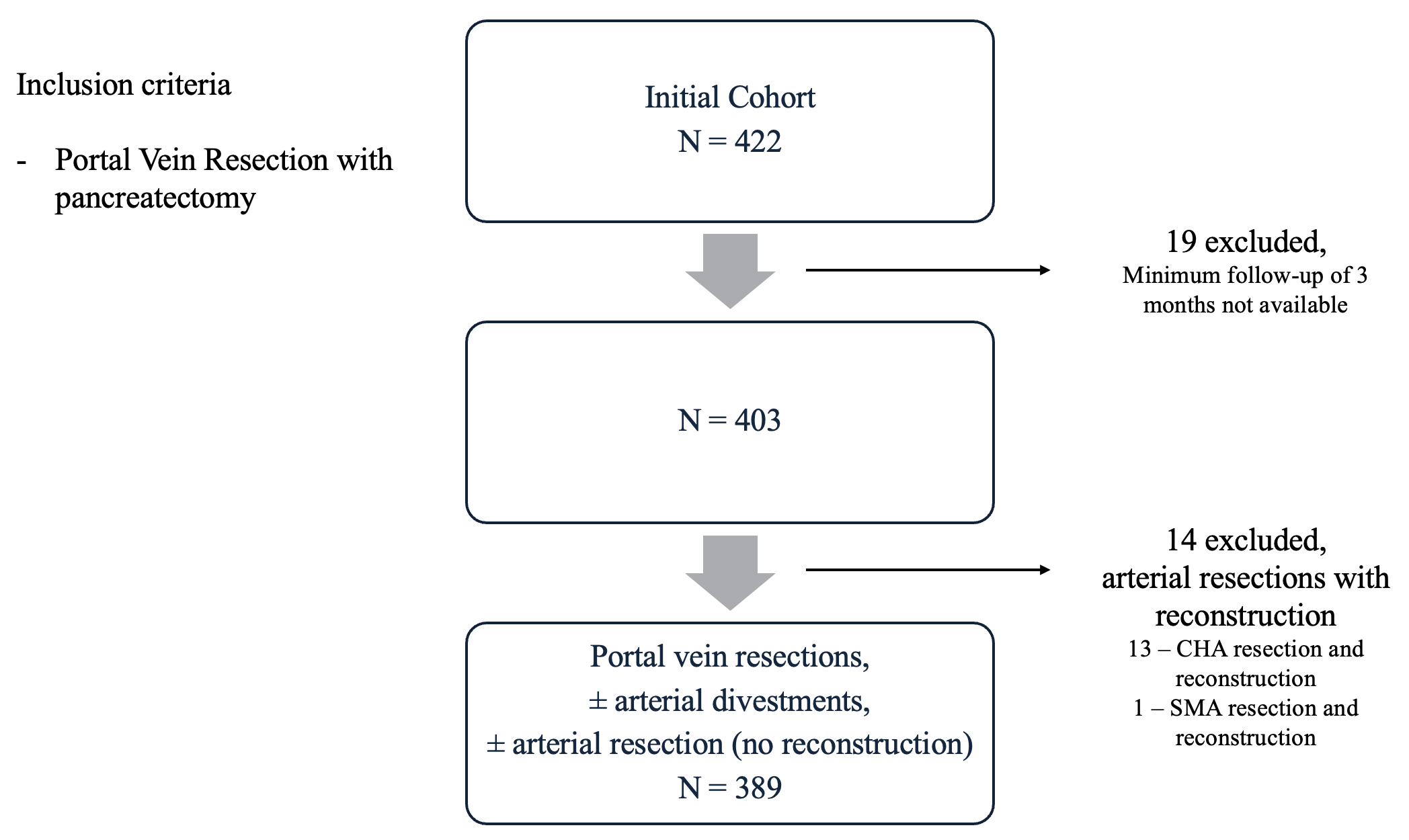


**Supplementary Table 1 - Clinico-demographic, preoperative and intra-operative parameters of the cohort.**

| **Clinical Parameters** | | **Total n = 389** |
| --- | --- | --- |
|  |  |  |
| **Gender** | **Male** | 230 (59.1%) |
|  | **Female** | 159 (40.9%) |
| **Age in years (mean ± SD)** |  | 56.1 ± 12.8 |
| **BMI in Kg/m^2^ (mean ± SD)** |  | 23.1 ± 3.9 |
| **Charlson’s Comorbidity Index** |  | 4 (2 - 8) |
| **ASA^a^ Score** | **1 + 2** | 309 (79.4%) |
|  | **3 + 4** | 80 (20.6%) |
| **Primary site of disease** | **Pancreas** | 321 (82.5%) |
|  | **Bile duct** | 32 (8.2%) |
|  | **Ampulla** | 19 (4.9%) |
|  | **Duodenum** | 13 (3.3%) |
|  | **Others** | 4 (1.0%) |
| **Type of primary disease** | **Adenocarcinoma** | 320 (82.3%) |
|  | **Neuroendocrine Tumour** | 31 (8.0%) |
|  | **Solid Pseudopapillary Neoplasm** | 15 (3.9%) |
|  | **Gastrointestinal Stromal Tumours** | 1 (0.3%) |
|  | **Non-malignant causes** | 22 (5.7%) |
| **Pancreatic Cancer** | **Yes** | 263 (67.6%) |
| **Preoperative Biliary Drainage** | **Yes** | 191 (49.1%) |
| **Radiological Resectablitiy** | **Resectable** | 151 (38.8%) |
|  | **Borderline Resectable** | 207 (53.2%) |
|  | **Locally Advanced** | 31 (8.0%) |
| **Preoperative Therapy** | **Yes** | 147 (37.9%) |
| **Preoperative Radiation** | **Yes** | 30 (7.7%) |
| **Planned Venous Resection** | **Yes** | 240 (61.7%) |
| **ISGPS^b^ Venous Resection Type** | **1** | 147 (37.8%) |
|  | **2** | 18 (4.6%) |
|  | **3** | 189 (48.6%) |
|  | **4** | 29 (7.5%) |
| **Vein Resection without Reconstruction** | | 6 (1.5%) |
| **Additional Organ Resection** | **Yes** | 58 (14.9%) |
| **Median Operative Blood Loss in ml (Median, IQR)** | | 600 (50 - 10500) |
| **Type of Surgery** | **Pancreatoduodenectomy** | 340 (87.4%) |
|  | **Distal Pancreato-splenectomy** | 35 (9.0%) |
|  | **Total Pancreatectomy** | 14 (3.6%) |
| **Pancreatic Texture** | **Soft** | 138 (37.0%) |
|  | **Non-soft** | 253 (63.0%) |
| **Main Pancreatic Duct** | **≤ 3 mm** | 125 (34.3%) |
|  | **> 3 mm** | 239 (65.7%) |
| **Post Pancreatectomy Haemorrhage** | **A** | 28 (7.2%) |
|  | **B** | 10 (2.6%) |
|  | **C** | 9 (2.3%) |
| **Postoperative Pancreatic Fistula** | **A** | 62 (15.9%) |
|  | **B** | 43 (11.1%) |
|  | **C** | 9 (2.3%) |
| **Delayed Gastric Emptying** | **A** | 79 (20.3%) |
|  | **B** | 47 (12.1%) |
|  | **C** | 15 (3.9%) |
| **Re-exploration** |  | 25 (6.4%) |

^a^ American Society of Anesthesiologists

^b^ International Study Group of Pancreatic Surgery

**Supplementary Table 2 – Centre-wise distribution of cases with reporting of the main outcome measures.**

| **Institute** | **Number of vein resections** | **Segmental vein resections (ISGPS type 3 or 4 or vein resection without reconstruction)** | **Major complications** | **30 day postoperative mortality** | **90 day postoperative mortality** |
| --- | --- | --- | --- | --- | --- |
| **Tata Memorial Hospital. Mumbai** | 116 | 86 (74.1%) | 53 (45.7%) | 3 (2.6%) | 10 (8.6%) |
| **Lakeshore Hospital, Ernakulam** | 66 | 30 (45.5%) | 35 (53.0%) | 1 (1.5%) | 6 (9.1%) |
| **Jawaharlal Institute of Postgraduate Medical Education and Research, Puducherry** | 42 | 23 (54.8%) | 10 (23.8%) | 1 (2.4%) | 1 (2.4%) |
| **Asian Institute of Gastroenterology, Hyderabad** | 37 | 14 (37.8%) | 5 (13.5%) | 1 (2.7%) | 1 (2.7%) |
| **Apollo Hospital, Bangalore** | 32 | 29 (90.6%) | 6 (18.6%) | 1 (3.1%) | 2 (6.3%) |
| **Amrita Institute of Medical Sciences, Kochi** | 20 | 9 (45.0%) | 6 (30.0%) | 1 (5.0%) | 2 (10.0%) |
| **Postgraduate Institute of Medical Education and Research, Chandigarh** | 22 | 12 (54.5%) | 3 (13.6%) | 2 (9.1%) | 2 (9.1%) |
| **Manipal Hospital/ Sakra Hospital, Bengaluru** | 19 | 12 (63.2%) | 4 (21.1%) | 0 | 0 |
| **Medanta Hospital, Gurugram** | 16 | 3 (18.8%) | 1 (6.25%) | 0 | 0 |
| **All India Institute of Medical Sciences, Jodhpur** | 14 | 3 (21.4%) | 3 (21.4%) | 1 (7.1%) | 1 (7.1%) |
| **Sanjay Gandhi Post Graduate Institute of Medical Sciences, Lucknow** | 5 | 2 (40.0%) | 1 (20.0%) | 0 | 0 |
| **Total** | 389 | 223 (57.3%) | 127 (32.6%) | 11 (2.8%) | 25 (6.4%) |

**Supplementary Table 3 – Factors associated with Failure to Rescue**

| **Clinical Parameters** | | **Failure to Rescue (n=127)** | | **p value** |
| --- | --- | --- | --- | --- |
|  |  | **No (n = 102)** | **Yes (n = 25)** |  |
|  |  |  |  |  |
| **Sex** | **Male** | 44 (43.1%) | 10 (40.0%) | 0.825 |
|  | **Female** | 58 (56.9%) | 15 (60.0%) |  |
| **Charlson Comorbidity Index > 4** | | 36 (35.3%) | 17 (68.0%) | *0.006* |
| **ASA Score^a^** | **1 + 2** | 72 (70.6%) | 17 (68.0%) | 0.811 |
|  | **3 + 4** | 30 (29.4%) | 8 (32.0%) |  |
| **Preoperative Biliary Drainage** | **Yes** | 46 (45.1%) | 17 (68.0%) | *0.047* |
| **Preoperative Therapy** | **Yes** | 43 (42.2%) | 14 (56.0%) | 0.264 |
| **Preoperative Radiation** | **Yes** | 11 (10.8%) | 5 (20.0%) | 0.309 |
| **Planned Venous Resection** | **Yes** | 67 (65.7%) | 15 (60.0%) | 0.644 |
| **ISGPS^b^ Venous Resection Type** | **1 + 2** | 43 (43.2%) | 4 (16.0%) | *0.020* |
|  | **3 + 4 + Others** | 59 (57.8%) | 21 (84.0%) |  |
| **Additional Organ Resection** | **Yes** | 23 (22.5%) | 7 (28.0%) | 0.603 |

^a^ American Society of Anesthesiologists

^b^ International Study Group of Pancreatic Surgery

p values significant at < 0.05 have been italicised.
